# The Late Arthur M. Barker, M. D.

**Published:** 1888-01

**Authors:** 


### *THE LATE ARTHUR M. BARKER, M. D.*

It is with profound regret, and a sense of loss to the medical profession, that the death of Dr. Arthur M. Barker is announced in these columns.

Born at Kendall, N. Y., 1850, he was educated in the public schools of this city, and received his medical degree in 1877. Shortly after his graduation, he received the appointment of House Physician at the Buffalo Medical and Surgical Dispensary—a position at that time affording valuable clinical experience.

Dr. Barker soon showed fitness for higher professional trusts. He was appointed one of the physicians to the Hospital of

Sisters of Charity. In '81 and '82, he held the office of Post-mortem Examiner, and for several years was the physician to the Buffalo Orphan Asylum, and also received the appointment of Lecturer on the Diseases of Children at the Buffalo Medical College.

He took an active interest in the various medical societies of the city, and was a valued member of the Buffalo Medical Club.

Of his excellences as a physician and surgeon, and as a man, the members of the profession have borne testimony in the expressions of both old and young at the largely-attended meeting of the Erie County Society.

Of his standing in the community, the eulogies which have appeared in the daily papers, illustrate how highly he was esteemed as an able physician and a good man: indeed, few physicians of his age have acquired so extensive a practice, and few have had so many warm friends.

In the full vigor of a splendid manhood, he was suddently cut off after about one week's illness; his death occurring on the sixth of December, the fatal affection being (probably) malignant endocarditis. It is is to be regretted that a post-mortem examination was not permitted.

